# HEARING INTERVENTION TO REDUCE COGNITIVE DECLINE: DESIGN AND FINDINGS OF THE ACHIEVE RANDOMIZED CONTROLLED TRIAL

**DOI:** 10.1093/geroni/igad104.0294

**Published:** 2023-12-21

**Authors:** Jennifer Deal, Nicholas Reed, Luigi Ferrucci

## Abstract

Epidemiologic data consistently link age-related hearing loss with accelerated cognitive decline and incident dementia in large, population-based studies. Mechanistic pathways that could underlie this association include the effects of distorted peripheral encoding of sound on cognitive load, changes to brain structure/function, and/or reduced social engagement and increased loneliness. Importantly, these pathways may be amenable to comprehensive hearing rehabilitative treatment including hearing assistive technologies (e.g., hearing aids, other integrated hearing assistive devices) and rehabilitative training. To date, however, there has never been a randomized trial that has investigated whether hearing loss treatment could reduce cognitive and other functional decline in older adults without dementia. The Aging and Cognitive Health Evaluation in Elders (ACHIEVE) trial is a multicenter randomized control trial of 977 cognitively intact adults with untreated, mild-to-moderate hearing loss aged 70-84 years at baseline (2018-19) to determine efficacy of best-practices hearing intervention to reduce cognitive decline over 3 years (Clinicaltrials.gov Identifier: NCT03243422). This session, on behalf of the ACHIEVE Collaborative Research Group, will describe the overall design of the ACHIEVE trial including details about recruitment and implementation of both the hearing (amplification, auditory rehabilitation) and successful aging health education control interventions. We will also present the primary findings of the study – the effect of the hearing intervention vs. control on 3-year cognitive decline – as well as the effect of the hearing intervention on several pre-specified outcomes important for the health of older adults, including social engagement, mood disorders and quality of life.

